# Diurnal Changes in the Transport Rates of Ureides, Amides, Cations, Anions, and Organic Acids Estimated by Xylem Sap Exudate and the Water Flow Rate of Soybean Plants

**DOI:** 10.3390/plants15040561

**Published:** 2026-02-11

**Authors:** Ryo Toyoda, Kyoko Higuchi, Akihiro Saito, Takuji Ohyama

**Affiliations:** 1 Laboratory of Biochemistry in Plant Productivity, Department of Agricultural Chemistry, Tokyo University of Agriculture, Setagaya-ku 156-8502, Japankhiguchi@nodai.ac.jp (K.H.); a3saito@nodai.ac.jp (A.S.); 2 Faculty of Agriculture, Niigata University, Niigata 950-2181, Japan

**Keywords:** allantoate, detached shoot, diurnal changes, soybean, transport rate, transpiration, xylem sap exudation rate, water flow rate

## Abstract

Ureides; allantoate, allantoin, and amides; asparagine, and glutamine are the N_2_ fixation products in soybean root nodules, and they are transported through xylem vessels. We estimated the transport rates of xylem constituents by multiplying nutrient concentrations by the water flow rate. Nodulated soybean plants were grown with an N-free solution under either 28 °C day/18 °C night or 28 °C day/28 °C night conditions, and diurnal changes in nutrient concentrations in xylem sap and transpiration rate were determined every 2 h. Under both conditions, xylem sap exudation rate and transpiration rate were high in light, and low, but not zero, in darkness. The sum of the xylem sap exudation rate and transpiration rate from detached shoots was almost the same as the water flow rate of intact plants at any time. All the N compounds exhibited a similar pattern: concentrations were high, but transport rates were lower at night. The proportions of N constituents were constant throughout the day and night. The composition and transport rate of xylem sap were not affected by night temperatures, except for cations. The results confirmed that the water flow rate and transport rate of xylem constituents can be estimated using detached roots and detached shoots.

## 1. Introduction

Soybean seeds contain high concentrations of protein and oil, and their world production increased from 278 million tons in 2014 to 398 million tons in 2024 (FAOSTAT). The world’s average yield of soybeans has also risen, from 2.61 t/ha in 2014 to 2.78 t/ha in 2024. Nitrogen (N) is the most crucial nutrient for plant growth and crop yield. Soybean plants depend on the N fixed by root nodules and the N absorbed by their roots. It has been established that the initial assimilation product is NH_4_^+^, which is derived either from the nitrogen fixed in nodules or from the NO_3_^−^ reduction in the roots and leaves [1]. However, the principal forms of N transport from the nodules and roots are different [1,2]. Tracer experiments with ^15^N_2_ revealed that the ammonia produced by nitrogen fixation in bacteroids is rapidly released to the cytosol of infected cells and is initially assimilated into the amide group of glutamine (Gln) by the enzyme glutamine synthetase [3,4]. Then, Gln and 2-oxoglutarate are metabolized to two molecules of glutamate by the enzyme glutamate synthase [1,5]. A major part of Gln is used for de novo urate synthesis in infected cells within the symbiotic region of the nodules [5,6]. Urate is transported from infected to adjacent uninfected cells, catabolized into allantoin and allantoate, and then transported to the shoot through xylem vessels [1,5,6,7]. Ureide-transporting leguminous plants are only found among tropical legumes, Phaseoleae, including *Glycine*, *Vigna*, and *Phaseolus* species [8]. Other legumes generally transport amides, primarily asparagine (Asn) [1]. It has been reported that ureides are the principal N transport compounds from soybean nodules, and that Asn, Gln, aspartate, glutamate, arginine, and other amino acids are also transported from the root nodules [9,10,11].

Allantoate and allantoin are produced in non-nodulated soybean roots [6,12]. However, their concentrations in the xylem sap in non-nodulated soybeans are approximately 10–30% of the sum of ureides, amides, and NO_3_^−^, and the proportion is much lower than that in the root nodules, which accounts for about 80–90% of all fixed N [5,6,7,12]. The mechanisms underlying why ureide synthesis is dominant in the nodules but not in the roots are not fully understood.

Some reports have shown [13] that N fixed in the nodules and N absorbed from the roots are differentially transported to the leaves and pods. Absorbed N, especially NO_3_^−^, is initially reduced in the roots or transported to the leaves and assimilated there; the assimilated N is then redistributed to the pods and seeds [13], while the fixed N is transported to both leaves and pods [13]. Excess N fertilizer can sometimes cause leaf overgrowth, but insufficient N is supplied to the pods.

Nutrients dissolved in soil water are absorbed by the epidermis and cortical cells of the roots, then cross the endodermis through the symplast pathway into the stele [1,5,14]. Then, the nutrients are unloaded from xylem parenchyma cells into the stele apoplast, and the dissolved nutrients are transported to the shoot via xylem vessels. This upward movement of the solution is driven by both transpiration from the leaves and root pressure [14]. The xylem sap collected from the cut stem depends only on the root pressure because of the lack of transpiration. The compositions of metabolites and inorganic ions in xylem sap obtained from the cut stem may reflect the actual compositions in intact plants. However, the composition or concentration of nutrients in xylem sap may change after shoot removal, especially long after cutting, due to a lack of photoassimilate transport from the leaves to the roots via the phloem.

Nutritional disorders, such as nutrient deficiencies or toxicities, cause reduced plant growth and lower crop yield and quality [14,15]. The diagnosis of nutritional disorders is crucial for optimizing crop growth, yield, and quality by ensuring adequate nutrient supply [14,15,16]. Determining the optimal forms, amounts, timing, and placement of inorganic and organic fertilizers can help avoid excess application, minimizing fertilizer cost and environmental impact [17]. Generally, the diagnosis of nutritional disorders is based on visible symptoms, plant analysis, and soil analysis [14,15]. Sometimes, a deficiency or excess of nutrients causes visible symptoms such as chlorosis, necrosis, or leaf deformation, as well as toxic symptoms on fruits [15]. Visible symptoms often occur under long-term deficiency or toxicity conditions, and consequently, crops cannot recover once the symptoms become apparent. In the plant analysis, mineral concentrations in leaves are determined by chemical analysis [15]. The procedures for the chemical analysis require significant labour and are time-consuming. Leaves are dried, ground into a powder, and then decomposed by chemical or heat digestion to solubilize the mineral elements in the plant samples. Instead, plant analysis using squeezed petiole juice or xylem sap is more convenient, as it requires no drying, grinding, digestion, or extraction [18,19,20,21,22,23,24,25,26]. Xylem sap collected from the cut stem, or juice squeezed from petioles or leaf blades, has been used to diagnose N, P, or micronutrient nutrition [20,21,22,23]. Based on the NO_3_^−^ concentrations in cucumber petiole juice, Roppongi proposed an optimum range [20]. A similar NO_3_^−^ diagnosis was reported for eggplants [23]. The concentrations of mineral elements, K, Ca, Mg, P, S, Zn, Fe, Mn, Cu, Mo, and Si in xylem sap collected from soybeans increase during the vegetative stage and then decrease during the reproductive stage [24]. A relative ureide method has been applied to field-grown soybeans to estimate the proportion of N derived from N_2_ fixation by the root nodules and N absorbed from the roots [25,26,27,28]. Ureide N concentrations are assumed to be N derived from N_2_ fixation, while the sum of the N concentrations of amides and NO_3_^−^ is the N derived from the roots [25,26,27,28].

Several methods have been used for collecting xylem sap from plants [29,30,31,32]. Xylem sap evacuated from a cut stem is monitored to investigate the nitrogen translocation forms of a wide variety of plants among dicotyledons, monocotyledons, and gymnosperms [30]. Gln and Asn are dominant in most species, but some plants contain citrulline or allantoic acid as the principal component in their xylem sap. Another method for xylem sap collection depends on the decapitation of shoots and the collection of the sap exudated from the cut end of the stem [25]. Xylem sap exudation from a cut stem depends only on the root pressure, because there is no transpiration after decapitation. So, this is called the “root pressure method”, which is the simplest way to collect xylem sap, and it can be easily applied to field experiments [25,26].

There has been debate over whether the nutrient concentration in xylem sap collected by the root pressure method matches the actual concentration in intact plants [29,33]. The concentrations of nutrients collected from the cut stem may differ from those in xylem sap from intact plants. However, it is difficult to answer this question because there is no non-invasive method for collecting actual xylem sap from an intact plant. Moreover, how best to estimate the flux or transport rate of nutrients from xylem sap concentrations and xylem sap exudation or transpiration rates remains an open question.

Yamamura et al. [33] found that the multiplication value of the ^33^P concentration in xylem sap and the water flow rate (xylem sap exudation rate from decapitated roots + the transpiration rate from detached shoots) was almost equivalent to the transport rate of ^33^P determined in intact shoots. The results support the conclusion that the Pi concentrations in xylem sap collected during the initial 1 h after cutting are the same as the actual Pi concentrations in intact plants. This method may be applicable to estimate nutrient transport rates for plant diagnosis. However, the xylem sap exudation rate and transpiration rate show diurnal fluctuations, so the question is posed of whether the water flow rate estimated by the sum of xylem sap exudation and the transpiration rate from the detached shoot is equivalent to that of the intact plants at any time of the day. In addition, it is not known whether night-time temperature affects xylem sap concentrations and the transport rate of major components.

In this research, we investigated diurnal changes in the concentrations of major N compounds (allantoate, allantoin, Asn, and Gln), cations (K, Mg, and Ca), anions (Pi and SO_4_), and organic acids (citrate and malate) in xylem sap obtained from nodulated soybean plants cultivated in N-free culture solution under controlled conditions. In addition, we calculated the transport rate of these nutrients using a new estimation method. We usually grow soybean plants under 28 °C for 16 h a day and 18 °C for 8 h at night [33,34]. During the night, a lack of light and low temperature may differentially affect the absorption and transport of nutrients and metabolites. So, we investigated the effects of night temperatures on nutrient concentrations and transport rates.

## 2. Results

The nodulated soybeans were grown with an N-free culture solution in a controlled chamber under either 28 °C day/18 °C night or 28 °C day/28 °C night conditions. The diurnal changes in nutrient concentrations in xylem sap and transpiration rate were determined every 2 h. In the experiment, under 28 °C day/18 °C night conditions, the water flow rate, calculated as the sum of the xylem sap exudation rate and the transpiration rate from the detached shoot, was compared with that of intact plants.

### 2.1. Comparison of the Sum of Xylem Sap Exudation Rate and Transpiration from the Detached Shoot and the Intact Plants Under 28 °C Day/18 °C Night Conditions

**Figure 1:**
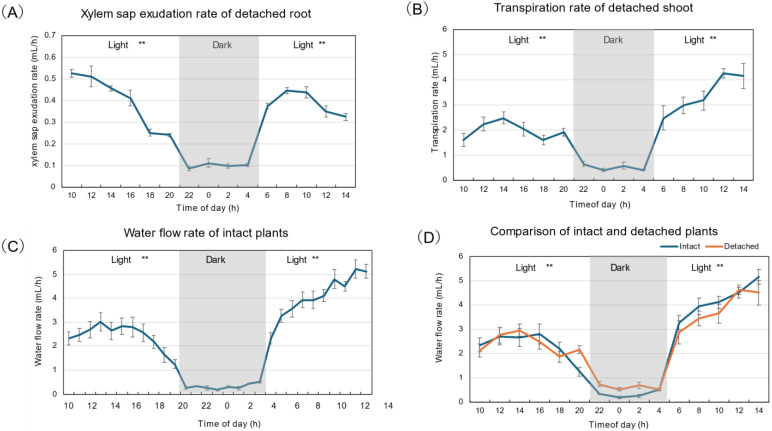
Diurnal changes in xylem sap exudation rate and transpiration rate of detached plants and intact plants under 28 °C day for 16 h from 5 AM to 9 PM /18 °C night for 8 h from 9 PM to 5 AM conditions. The experiment began at 10 AM on 30 DAP and ended at 3 PM (15 AM) on 31 DAP. (**A**) Xylem sap exudation rate from detached root. (**B**) Transpiration rate of detached shoot. (**C**) Transpiration rate of intact plants. (**D**) Comparison of the water flow rate of intact plants (transpiration rate) and the detached plants (sum of transpiration of xylem sap exudation rate and the transpiration rate in the detached shoot). The transpiration rate of intact plants in this figure is the average over every two hours, as for the detached plants. Averages ± standard errors. *n* = 3. The grey background shows night-time, and the white background shows day-time. ** indicate statistical differences at *p* < 0.01 between the 1st light period (*n* = 18:6 time points and 3 replications) and dark period (*n* = 12: 4 time points and 3 replications), and the dark period and 2nd light period (*n* = 15: 5 time points and 3 replications) based on Student’s *t*-test (**A**,**B**,**D**), or Welch’s *t*-test (**C**) for intact plants; the 1st light period (*n* = 33: 11 time points and 3 replications) and dark period (*n* = 24: 8 time points and 3 replications), and 2nd light period (*n* = 30: 10 time points and 3 replications).

Figure 1A shows the xylem sap exudation rate from the decapitated root. The xylem sap exudation rate gradually decreased from 0.5 mL/h to 0.2 mL/h during the first light period. During the dark period, the xylem sap exudation rate was significantly lower (*p* < 0.01), but remained consistently at about 0.1 mL/h. The exudation rate recovered to about 0.4 mL/h at 6 AM during the second light period. The exudation rate was highest around 8 AM and 10 AM and decreased thereafter. The transpiration rate from the detached shoot was always higher than the xylem sap exudation rate, reaching a maximum of about 2.5 mL/h around 2 PM, then decreasing during the first light period (Figure 1B). The transpiration rate during the dark period was constant at about 0.5 mL/h and lower than during the first (*p* < 0.01) and second light periods (*p* < 0.01). The transpiration rate recovered rapidly at 6 AM after a transition from dark to light conditions and increased thereafter. Figure 1C shows the diurnal changes in water flow rate from intact plants cultivated simultaneously with detached plants. The weight of the plants in the bottle was measured every hour, and transpiration was calculated as the loss in weight. The transpiration rate increased from 2.3 mL/h at 10 AM to 3 mL/h at 1 PM, and decreased to 1 mL/h at the end of the first light period. During the dark period, transpiration rates were significantly lower than during light periods (*p* < 0.01), ranging from 0.1 to 0.5 mL/h. The transpiration rate of intact plants increased rapidly after the second light period began and continued until 2 PM. The sum of the xylem sap exudation rate (Figure 1A) and the transpiration rate from the detached shoot (Figure 1B) was similar to the transpiration rate from the intact plants (Figure 1D). This indicates that the transpiration rate estimated from the xylem sap exudation rate plus that from the detached shoot can be used as the transpiration rate in intact plants. This method helps estimate the transpiration rate of soybeans grown in the field, as it is difficult to measure the plants’ actual transpiration rate.

### 2.2. Diurnal Changes in the Concentration and Transport Rate of Major N Constituents, Cations, Anions, and Organic Acids in Xylem Sap Under 28 °C Day/18 °C Night Conditions

**Figure 2:**
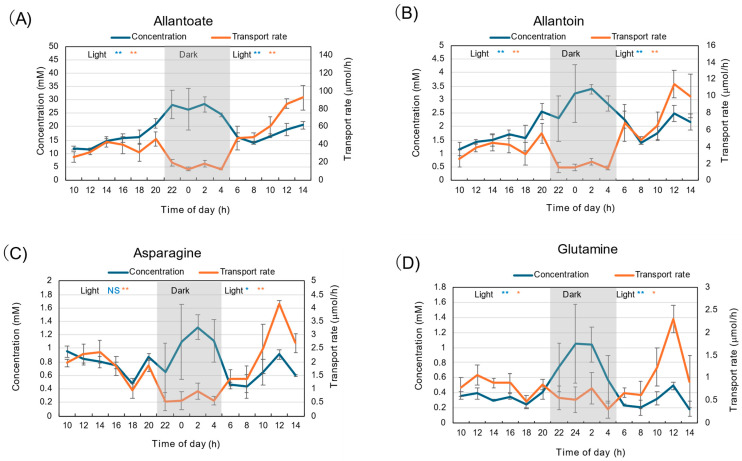
Diurnal changes in concentration and transport rate of major N compounds in xylem sap under 28 °C day/18 °C night conditions. (**A**) Allantoate, (**B**) Allantoin, (**C**) Asparagine, (**D**) Glutamine. Averages ± standard errors. *n* = 3. The grey background shows night-time from 9 PM to 5 AM, and the white background shows day-time from 5 AM to 9 PM. *, ** and NS after light indicate statistical differences between the 1st light period (*n* = 18) and dark period (*n* = 12), and the dark period and 2nd light period (*n* = 15) at *p* < 0.05, *p* < 0.01, and not significant by Student’s *t*-test (Blue: concentration, Red: transport rate).

Diurnal changes in the concentrations of major N constituents, allantoate, allantoin, Asn, and Gln, under 28 °C day/18 °C night conditions are shown in Figure 2. In the figure, the transport rate is also shown, with the concentration multiplied by the water flow rate estimated as the sum of the xylem sap exudation rate and the transpiration rate from the detached shoot. The concentration of allantoate was highest among all N constituents measured (Figure 2A). The concentration was 12 mM at 10 AM and increased to 20 mM at the end of the first light period. The concentration of allantoate during the dark period was about 25–30 mM, significantly higher than the light periods (*p* < 0.01), and then decreased to 15 mM at the beginning of the second light period. The trends were similar for allantoin (Figure 2B), Asn (Figure 2C), and Gln (Figure 2D), although the concentrations of allantoin, Asn, and Gln were lower than that of allantoate.

The transport rate of allantoate was about 30–40 μmol/h during the first light period and quickly decreased to 10–20 μmol/h during the dark period. The allantoate transport rate recovered to 50 μmol/h at the beginning of the second light period, and then it increased thereafter. The trends were similar among all constituents, including allantoin, Asn, and Gln.

**Figure 3:**
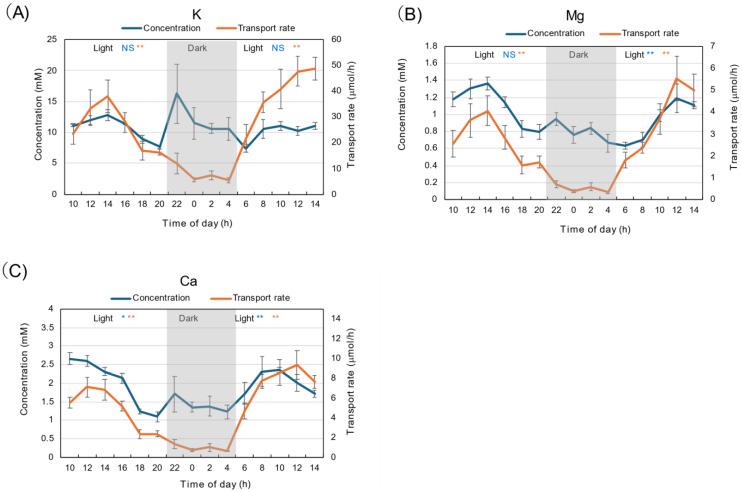
Diurnal changes in concentration and transport rate of major cations in xylem sap under 28 °C day/18 °C night conditions. (**A**) K, (**B**) Mg, (**C**) Ca. Averages ± standard errors. *n* = 3. The grey background shows night-time from 9 PM (21 AM) to 5 AM, and the white background shows day-time from 5 AM to 9 PM (21 AM). *, ** and NS after light indicate statistical differences between the 1st light period (*n* = 18) and dark period (*n* = 12), and the dark period and 2nd light period (*n* = 15) at *p* < 0.05, *p* < 0.01, and not significant by Student’s *t*-test (Blue: concentration, Red: transport rate).

Different from the concentration of N constituents, the diurnal changes in K concentration remained relatively constant at about 10–15 mM throughout the light and dark periods (Figure 3A). The average concentration during the dark period was not statistically different from the first and second light periods. The concentrations of Mg (Figure 3B) and Ca (Figure 3C) during the dark period were about half of those during the light period. The transport rates of K, Mg, and Ca were significantly lower (*p* < 0.01) during the dark period than during the light period. The patterns of the changes in the transport rates of K, Mg, and Ca were similar to the transpiration rate shown in Figure 1D.

**Figure 4:**
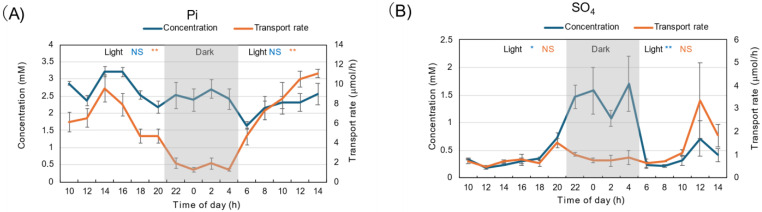
Diurnal changes in concentration and transport rate of major anions in xylem sap under 28 °C day/18 °C night conditions. (**A**) Pi, (**B**) SO_4_. Averages ± standard errors. *n* = 3. The grey background shows night-time from 9 PM (21 AM) to 5 AM, and the white background shows day-time from 5 AM to 9 PM (21 AM). *, **, and NS after light indicate statistical differences between the 1st light period (*n* = 18) and dark period (*n* = 12), and the dark period and 2nd light period (*n* = 15) at *p* < 0.05, *p* < 0.01, and not significant by Student’s *t*-test (Blue: concentration, Red: transport rate).

The diurnal changes in Pi concentration in xylem sap were relatively constant, about 2–3 mM, both during the light and dark periods (Figure 4A). The transport rate of Pi was high during the light period, peaking at 2 PM, and decreased thereafter. The Pi concentration was as low as 0.5 mM during the dark period and increased during the second light period. The SO_4_ concentration was about 0.3 mM during the first light period, increased to 1.5 mM during the dark period, then decreased to 0.2 mM at the start of the second light period (Figure 4B). Due to high SO_4_ concentrations during the dark period, transport rates were relatively constant at about 1 μmol/h, irrespective of the light or dark period, and were not statistically significant.

**Figure 5:**
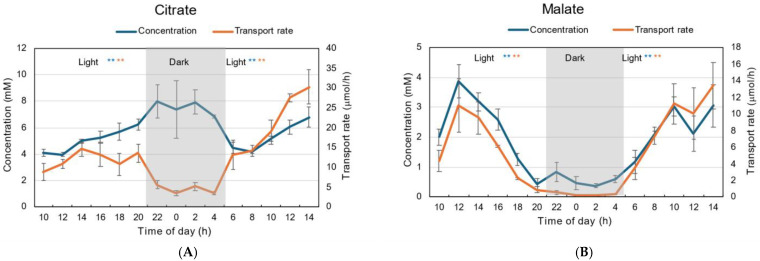
Diurnal changes in the concentration and transport rate of major organic acids in xylem sap under 28 °C day/18 °C night conditions. (**A**) Malate, (**B**) Citrate. Averages ± standard errors. *n* = 3. The grey background shows night-time from 9 PM (21 AM) to 5 AM, and the white background shows day-time from 5 AM to 9 PM (21 AM). * and ** after light indicate statistical differences between the 1st light period (*n* = 18) and dark period (*n* = 12), and dark period and 2nd light period (*n* = 15) at *p* < 0.05, and *p* < 0.01, by Student’s *t*-test (Blue: concentration, Red: transport rate).

The patterns of diurnal changes in citrate concentration were quite different from those in malate (Figure 5). The concentration of citrate increased from 4 mM to 6 mM during the first light period, and remained at the highest concentration of about 8 mM during the dark period (Figure 5A). At the beginning of the second light period, the citrate concentration decreased to 4 mM and then gradually increased to 7 mM. The transport rate of citrate during the dark period was about 3–5 mM, significantly lower than during the first and second light periods (*p* < 0.01), similar to the changes in transpiration rate (Figure 1D).

The malate concentration was the highest at 12 AM, about 4 mM, then decreased to 0.5 mM at the end of the first light period (Figure 5B). The malate concentration was about 0.5 mM during the dark period and increased during the second light period. The changes in malate transport rates show a pattern similar to that of the concentration. The transport rate was the highest, about 10 μmol/h, at 12 AM, and decreased during the first light period. The transport rates of malate were very low during the dark period, then they increased in the second light period.

### 2.3. Diurnal Changes in pH of Xylem Sap Collected from Detached Roots Under 28 °C Day/18 °C Night Conditions

**Figure 6:**
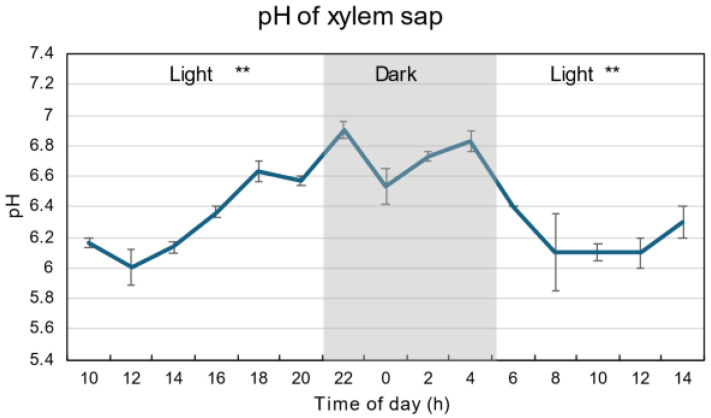
Diurnal changes in the pH of xylem sap under 28 °C day/18 °C night conditions. The grey background shows night-time from 9 PM (21 AM) to 5 AM, and the white background shows day-time from 5 AM to 9 PM (21 AM). ** indicate statistical differences at *p* < 0.01 between the 1st light period (*n* = 18) and dark period (*n* = 12), and then dark period and 2nd light period (*n* = 15) based on Student’s *t*-test.

Figure 6 shows the diurnal changes in the pH of the xylem sap. The pH during the dark period was about 6.6 to 6.9, and was significantly higher than that in the first and second light periods (*p* < 0.01), which were about 6.0 to 6.6.

### 2.4. Diurnal Changes in the Cumulative Concentration, N Transport Rate, and N Distribution of Allantoate, Allantoin, Asn, and Gln in Xylem Sap Under 28 °C Day/18 °C Night Conditions

**Figure 7:**
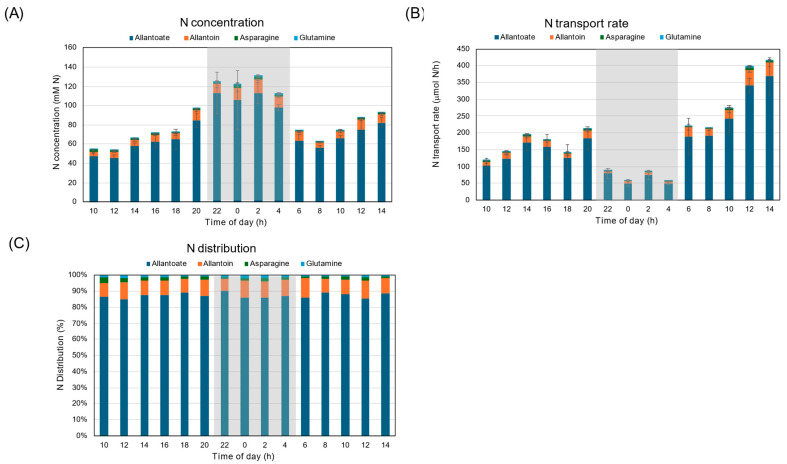
Comparison of diurnal changes in the cumulative concentrations and transport rate of allantoate, allantoin, and asparagine of soybean plants under 28 °C day/18 °C night conditions. (**A**) N concentration, (**B**) N transport rate, (**C**) distribution of N. Averages ± standard errors. *n* = 3. The grey background shows night-time from 9 PM to 5 AM, and the white background shows day-time from 5 AM to 9 PM.

Figure 7A shows the diurnal changes in the cumulative concentrations of allantoate, allantoin, Asn, and Gln in xylem sap. The sum of the major N constituents in soybean xylem sap was about 50 mM N at 10 AM, and it increased to 100 mM at the end of the first light period. The total N concentration was about 120 mM during the dark period, then returned to 70 mM at the beginning of the second light period, and then reached 90 mM at 14 AM. The N transport rate increased from 100 to 200 μmol N/h during the first light period, then decreased to 50–100 μmol N/h during the dark period (Figure 7B). Then the N transport rate increased to 200 μmol N/h at the beginning of the second light period, then increased to 400 μmol N/h at the end of the second light period. The percentage distributions of N compounds in xylem sap were consistent during the experimental period (Figure 7C). Allantoate accounted for about 85–90% of total N and did not change during the first light, dark, and the second light periods. Allantoin accounted for about 8.4–11% of total N, and Asn and Gln accounted for 1.3–3.4% and 0.7–1.7% each.

### 2.5. Relationships Between the Water Transport Rate, Concentration, and Transport Rate of Each Constituent in Xylem Sap Under 28 °C Day/18 °C Night Conditions

Figure S1 shows the relationships between the water flow rate, calculated as the xylem exudation rate plus the transpiration rate from the detached shoot, and the transport rate of each constituent. The correlation coefficients for the transport rates of allantoate (r = 0.933), allantoin (r = 0.905), and Asn (r = 0.868) were relatively high, whereas that for Gln (r = 0.691) was low. The correlation coefficients of cations, K (r = 0.964), Mg (r = 0.926), and Ca (r = 0.938) were high. The correlation was also high in Pi (r = 0.943), but low in SO_4_ (r = 0.543). Positive correlations were observed in citrate (r = 0.934) and malate (r = 0.883).

The relationships between the water transport rate and the concentrations of each constituent are shown in Figure S2. Weak negative correlations were observed among Gln, allantoate, Asn, SO_4_, and citrate, whereas weak positive correlations were shown among malate and Ca. No correlation was observed for allantoin, K, Mg, and Pi.

### 2.6. Comparison of the Sum of Xylem Sap Exudation Rate and Transpiration from the Detached Shoots and the Intact Plants Under 28 °C Day/28 °C Night Conditions

**Figure 8:**
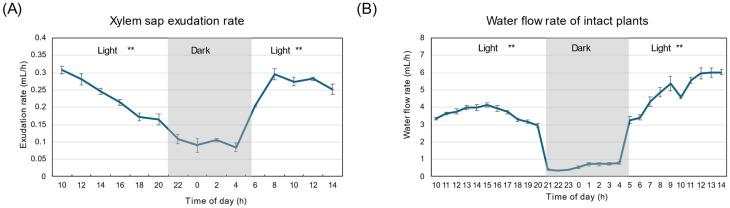
Diurnal changes in the xylem sap exudation rate and transpiration rate of soybean plants under 28 °C day/28 °C night conditions. (**A**) Xylem sap exudation rate, (**B**) transpiration rate of intact plants. Averages ± standard errors. *n* = 3. The grey background shows night-time from 9 PM (21 AM) to 5 AM, and the white background shows day-time from 5 AM to 9 PM (21 AM). ** indicate statistical differences at *p* < 0.01 between the 1st light period (*n* = 18) and dark period (*n* = 12), and dark period and 2nd light period (*n* = 15) based on Student’s *t*-test (**A**) or Welch’s *t*-test for intact plants (**B**); the 1st light period (*n* = 33) and dark period (*n* = 24), and 2nd light period (*n* = 30).

Figure 8A shows the xylem sap exudation rate from the detached roots under 28 °C day/28 °C night conditions. The xylem sap exudation rate decreased from 0.3 mL/h to 0.15 mL/h during the first light period, similar to that under 28 °C day/18 °C night conditions (Figure 1A). During the dark period, the xylem sap exudation rate was significantly lower than the first and the second light periods (*p* < 0.01), about 0.1 mL/h, but the xylem sap consistently exudated. The exudation rate recovered to about 0.2 mL/h at 6 AM during the second light period. The exudation rate was highest around 8 AM and 10 AM. Figure 8B shows the diurnal changes in water flow rate from the intact plants cultivated simultaneously with detached plants. The water flow rate increased from 3.3 mL/h at 10 AM to 4 mL/h at 9 PM and decreased to 3.0 mL/h at the end of the first light period. During the dark period, transpiration rates decreased to about 0.3–0.8 mL/h, which were significantly lower than those in the first and second light periods (*p* < 0.01). The transpiration rate of intact plants increased rapidly after the second light period started and increased until 2 PM.

### 2.7. Diurnal Changes in the Concentration and Transport Rate of Major N Constituents, Cations, Anions, and Organic Acids in Xylem Sap Under 28 °C Day/28 °C Night Conditions

**Figure 9:**
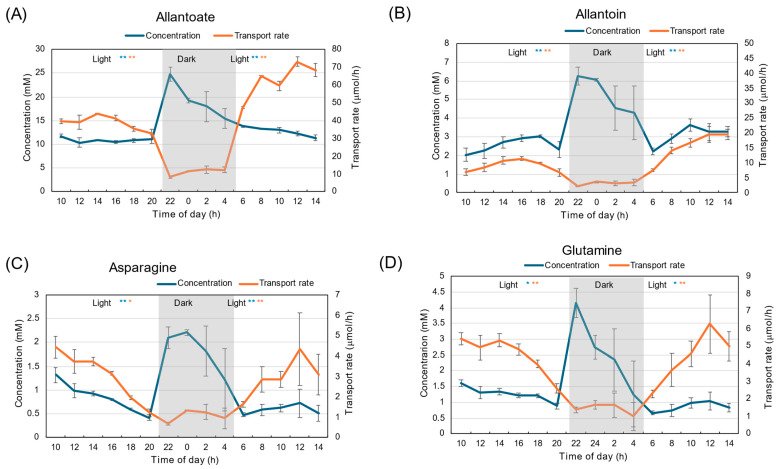
Diurnal changes in concentration and transport rate of major N compounds in xylem sap under 28 °C day/28 °C night conditions. (**A**) Allantoate, (**B**) Allantoin, (**C**) Asparagine, (**D**) Glutamine. Averages ± standard errors. *n* = 3. The grey background shows night-time from 9 PM (21 AM) to 5 AM, and the white background shows day-time from 5 AM to 9 PM (21 AM). * and ** after light indicate statistical differences between the 1st light period (*n* = 18) and dark period (*n* = 12), and the dark period and 2nd light period (*n* = 15) at *p* < 0.05, and *p* < 0.01 by Student’s-*t*-test (Blue: concentration, Red transport rate).

Diurnal changes in the concentration of major N constituents allantoate, allantoin, Asn, and Gln under 28 °C day/28 °C night conditions are shown in Figure 9. The concentration of allantoate was highest among all N constituents measured (Figure 9A). The allantoate concentration was 12 mM at 10 AM, and remained constant until 8 PM. It suddenly increased to 25 mM at 10 PM, the beginning of the dark period. The concentration of allantoate gradually decreased to 15 mM during the dark period, and then it continued to decrease to 12 mM at 2 PM in the second light period. The higher concentration observed during the dark period was similar in allantoin (Figure 9B), Asn (Figure 9C), and Gln (Figure 9D).

The transport rate of allantoate was approximately 40 μmol/h during the first light period and decreased to 10 μmol/h at the beginning of the dark period. Then the allantoate transport rate recovered during the second light period, to 50 μmol/h, and increased to 70 μmol/h at 12 AM. The trends were similar among all constituents, including allantoin, Asn, and Gln.

**Figure 10:**
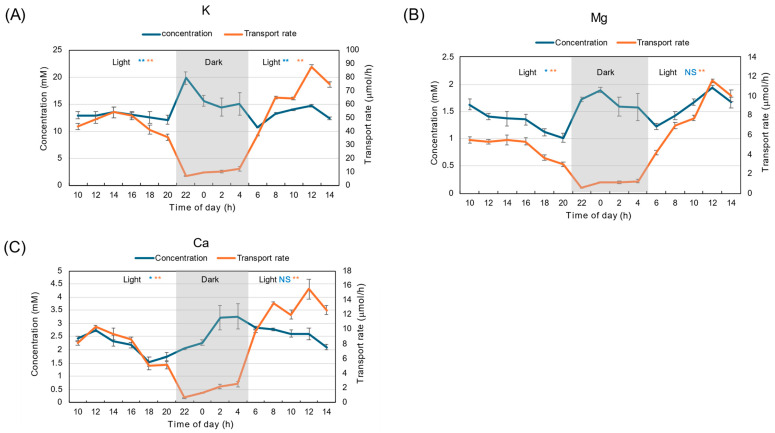
Diurnal changes in concentration and transport rate of major cations in xylem sap under 28 °C day/28 °C night conditions. (**A**) K, (**B**) Mg, (**C**) Ca. Averages ± standard errors. *n* = 3. The grey background shows night-time from 9 PM (21 AM) to 5 AM, and the white background shows day-time from 5 AM to 9 PM (21 AM). *, ** and NS after light indicate statistical differences between the 1st light period (*n* = 18) and dark period (*n* = 12), and dark period and 2nd light period (*n* = 15), at *p* < 0.05, *p* < 0.01, and not significant by Student’s *t*-test (Blue: concentration, Red: transport rate).

Different from the diurnal changes in K concentration under 28 °C day/18 °C night conditions (Figure 3A), the diurnal changes in K concentration under 28 °C day/28 °C night conditions significantly increased to 20–15 mM during the dark periods, compared with the first light period, about 13 mM (Figure 10A). On the other hand, the concentrations of Mg (Figure 10B) and Ca (Figure 10C) during the dark period were only slightly increased compared with those during the first light period (*p* < 0.05), and were not significantly different compared with the second light period. The transport rates of K, Mg, and Ca were significantly lower during the dark period compared with the light periods (*p* < 0.01). The patterns of the changes in the transport rates of K, Mg, and Ca were similar to the water flow rate shown in Figure 8B.

**Figure 11:**
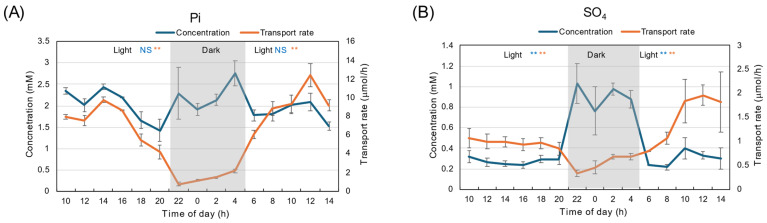
Diurnal changes in concentration and transport rate of major anions in xylem sap under 28 °C Day/28 °C Night conditions. (**A**) Pi, (**B**) SO_4_. Averages ± standard errors. *n* = 3. The grey background shows night-time from 9 PM (21 AM) to 5 AM, and the white background shows day-time from 5 AM to 9 PM (21AM). ** and NS after light indicate statistical differences between the 1st light period (*n* = 18) and dark period (*n* = 12), and dark period and 2nd light period (*n* = 15) at *p* < 0.01, and not significant by Student’s *t*-test (Blue: concentration, Red: transport rate).

The diurnal changes in Pi concentration in xylem sap under 28 °C day/28 °C night conditions were relatively constant, ranging from 1.5 to 2.5 mM during both light and dark periods (Figure 11A). The transport rate of Pi was significantly higher during the light period than the dark period (*p* < 0.01), peaking at 2 PM and decreasing thereafter. The Pi transport rate was as low as 1–2 μmol/h during the dark period, and the concentration increased during the second light period. The SO_4_ concentration was about 0.3 mM during the first light period, increased to 1.0 mM during the dark period, then decreased to 0.2 mM at the start of the second light period (Figure 11B). During the dark period, transport rates decreased from 1 to 0.5 μmol/h.

**Figure 12:**
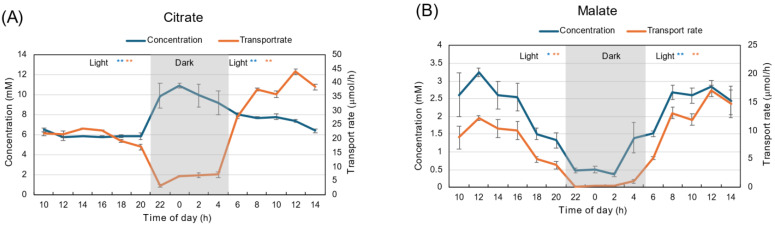
Diurnal changes in concentration and transport rate of major organic acids in xylem sap under 28 °C day/28 °C night conditions. (**A**) Citrate, (**B**) malate. Averages ± standard errors. *n* = 3. The grey background shows night-time from 9 PM (21 AM) to 5 AM, and the white background shows day-time from 5 AM to 9 PM (21 AM). * and ** after light indicate statistical differences between the 1st light period (*n* = 18) and dark period (*n* = 12), and the dark period and 2nd light period (*n* = 15) at *p* < 0.05, and *p* < 0.01 by Student’s *t*-test (Blue: concentration, Red: transport rate).

The patterns of diurnal changes in the concentrations of citrate and malate were quite different under 28 °C day/28 °C night conditions (Figure 12), as were those under 28 °C day/18 °C night conditions (Figure 5). The citrate concentration remained at 6 mM during the first light period and increased to 10 mM in the dark period (Figure 12A). At the beginning of the second light period, the citrate concentration gradually decreased to 6 mM. The transport rate of citrate during the dark period was about 5 μmol/h, and was significantly lower than during the first and second light periods (*p* < 0.01), similar to the changes in transpiration rate (Figure 8B).

The malate concentration was highest at 12 AM, about 3 mM, then decreased to 1.3 mM at the end of the first light period (Figure 12B). The malate concentration was about 0.5 mM during the dark period and increased during the second light period. The changes in malate transport rates show a pattern similar to that of concentration. The transport rate was the highest, about 10 μmol/h, at 12 AM, and decreased during the first light period. The transport rates of malate were very low during the dark period, then they increased in the second light period.

### 2.8. Diurnal Changes in the Cumulative Concentration, N Transport Rate, and N Distribution of Allantoate, Allantoin, Asn, and Gln in Xylem Sap Under 28 °C Day/28 °C Night Conditions

**Figure 13:**
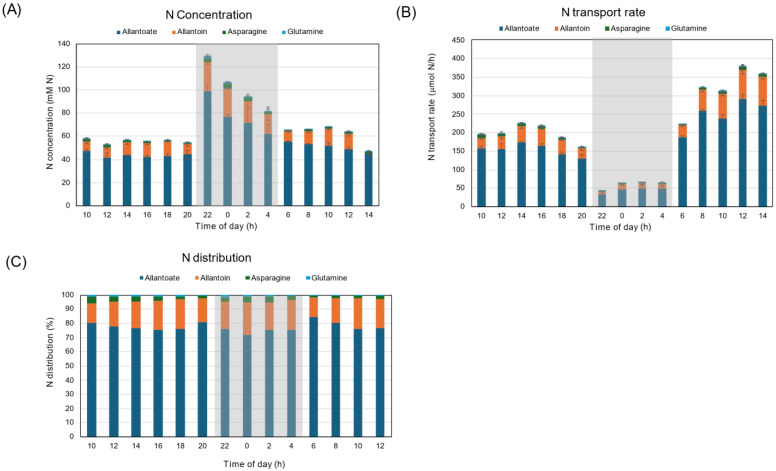
Comparison of diurnal changes in the concentrations and transport rate of allantoate, allantoin, and asparagine in soybean plants under 28 °C day/28 °C night conditions. (**A**) N concentration, (**B**) N transport rate, (**C**) distribution of N. Averages ± standard errors. *n* = 3. The grey background shows night-time from 9 PM (21 AM) to 5 AM, and the white background shows day-time from 5 AM to 9 PM (21 AM).

Figure 13A shows the diurnal changes in the cumulative concentrations of allantoate, allantoin, Asn, and Gln in xylem sap under 28 °C day/28 °C night conditions. The sum of the major N constituents in soybean xylem sap was about 50–60 mM N during the first light period, and increased to 120 mM at the beginning of the dark period. The total concentration decreased to 80 mM N during the dark period, then rose to 60 mM N at the start of the second light period. The N transport rate was about 200 μmol N/h during the first light period, then decreased to 50 μmol N/h during the dark period (Figure 13B). Then the N transport rate increased to 200 μmol N/h at the start of the second light period and to 360 μmol N/h at the end of the second light period. The percentage distributions of N compounds in xylem sap were consistent during the experimental period (Figure 13C). Allantoate accounted for about 75–80% of total N and did not change during the first light, dark, or second light periods. Allantoin accounted for about 14–23% of total N, Asn (1.5–4.6%), and Gln (0.5–1.5%).

### 2.9. Relationships Between the Transport Rate and Concentration of Each Constituent in Xylem Sap and the Water Flow Rate Under 28 °C Day/28 °C Night Conditions

Figure S3 shows the relationships between the water flow rate in the intact plants and the transpiration rate of each constituent. The correlation coefficients for the transport rates of allantoate (r = 0.980) and allantoin (r = 0.944) were very high, and those for Asn (r = 0.770) and Gln (r = 0.854) were positively correlated. The correlation coefficients of cations, K (r = 0.987), Mg (r = 0.957), and Ca (r = 0.957) were high. The correlation was also high in Pi (r = 0.950) and SO_4_ (r = 0.953). A positive correlation was also observed for citrate (r = 0.903) and malate (r = 0.864).

The relationships between the water flow rate and the concentration of each constituent are shown in Figure S4. Allantoate, allantoin, Asn, and Gln showed negative correlations. K, Mg, Ca, and Pi showed no correlations. On the other hand, SO_4_ and citrate showed weak negative correlations, and malate showed a positive correlation with water transport rate.

## 3. Discussion

### 3.1. Comparison of the Water Flow Rate of Intact Plants and That Estimated by the Xylem Sap Exudation Rate and Transpiration Rate from Detached Shoots

It is not easy to measure the actual transpiration rate in field-grown soybean plants. We compared the diurnal changes in the transpiration rate of intact plants grown in a glass bottle with the sum of the xylem sap exudation rate and the transpiration rate from the detached shoot under controlled conditions. As shown in Figure 1D, both values were nearly identical through the light and dark periods. Therefore, we concluded that this method helps in estimating soybean transpiration rates in the field. It was reported that the multiplied value of the ^33^P concentration in the xylem sap and the transpiration rate are equivalent to the transport rate of ^33^P in the intact shoots [33]. This result indicates that the ^33^P concentration in xylem sap collected from detached roots is approximately the same as that in intact plants. The P transport rate was measured in soybean plants grown in soil at R1, R5, and R7 stages by this method [33].

### 3.2. Diurnal Changes in the Concentration and Transport Rate of N Constituents

Because soybean plants were grown with an N-free solution in this experiment, all N originated from N_2_ fixation. The sum of the N transport rates of allantoate, allantoin, Asn, and Gln was considered to be the transport rate of fixed N, because these compounds are the principal N transport form from nodules. It was reported that waterlogging or changing the gas phase to N_2_ or to a mixture of Ar and O_2_ caused a rapid depression of N_2_ fixation, and the concentrations of Gln and ureides decreased rapidly [35]. This result indicates that a significant part of Gln and ureides in xylem sap is derived from N_2_ fixation.

Either under 28 °C day/18 °C night conditions (Figure 7B) or under 28 °C day/28 °C night conditions (Figure 13B) in this experiment, the N transport rate during the dark period was much lower than that under the light conditions. This phenomenon might be due to a decrease in transpiration rate and a reduction in N_2_ fixation activity. The N concentration during the dark period was higher than during the light period (Figure 7A and Figure 13A), suggesting that the sudden decrease in transpiration rate might reduce water supply and that N_2_ fixation products were condensed. Among N forms, allantoate was the most abundant, about 80–90% of total N, and allantoin accounted for 10–20%, with a small portion of Asn and Gln (Figure 7C and Figure 13C). The percentage remained constant during light and dark periods, indicating that day/night conditions did not affect the assimilation of fixed ammonia or the synthesis of ureides in nodules. The ureide-N percentage of total N in 11 soybean cultivars ranges from 72.5% to 82.5% under natural conditions in a greenhouse [28]. In this report, the diurnal changes in xylem sap exudation rates were analyzed as low, but the N concentration in xylem sap increased during the night. Similar results were reported for the accumulation of ureide-N and amino-N during a dark period in cowpea plants, although N_2_ fixation activity decreased under dark conditions [36].

### 3.3. Diurnal Changes in the Concentration and Transport Rate of Cations

The diurnal changes in the transport rates of K, Mg, and Ca exhibited similar patterns: they were highest at 14 AM, decreased during the light period, and were lower in the dark than in the light period (Figure 3). The diurnal fluctuations were similar to those in the transpiration rate (Figure 1C). Different from N compounds, the concentrations of K, Mg, and Ca under dark conditions were not higher than during the light period under 28 °C day/18 °C night conditions. Under 28 °C day/28 °C night conditions, changes in transport rates were similar, but the concentrations of K, Mg, and Ca were higher during the dark period than during the light period. The absorption rate of these cations declines at low temperatures, so a night temperature of 18 °C might affect their absorption and transport rates. Low night-time temperatures might affect the loading and unloading of cations in the xylem.

### 3.4. Diurnal Changes in the Concentration and Transport Rate of Anions

The fluctuations in Pi transport rate resembled those of cations and transpiration; Pi transport rate was lower in the dark than in the light. However, the transport rate of SO_4_ was relatively constant during dark and light periods under 28 °C day/18 °C night conditions. The concentration of Pi was steady throughout the light and dark periods, but the concentration of SO_4_ was much higher during the dark period than during the light periods. Yamamura et al. [33] reported that the ^33^P absorption rate from a culture solution is almost the same under light and dark conditions. In addition, the ^33^P absorption rate in the decapitated roots was similar to that of the intact plants under light. These results indicated that P absorption in soybean roots is not affected by light/dark conditions or by the lack of evapotranspiration for several hours. Unlike ^33^P absorption, ^33^P transport from the roots to the shoots is significantly lower in the dark than in the light, although a small amount of ^33^P reaches the shoot tips under both conditions. In the previous study [34], it was reported that Pi absorption rates increase with higher Pi concentrations in the culture solution during 3 days of 0–500 μM Pi treatments. Pi concentrations in xylem sap increase only from 0 to 50 μM Pi conditions in culture solution, and remain relatively constant at higher Pi concentrations up to 500 μM. These results indicated that Pi absorption from the culture solution and subsequent P transport from roots to shoots might be differentially regulated by external Pi concentrations.

### 3.5. Diurnal Changes in the Concentration and Transport Rate of Organic Acids

Citrate and malate are the major organic acids in soybean xylem sap; however, the changes in their transport rates and concentrations were quite different between under 28 °C day/18 °C night conditions (Figure 5) and 28 °C day/28 °C night conditions (Figure 12). The transport rate of citrate was lower in the dark period than in the light period, while the concentration was higher in the dark period, as shown for N compounds. A unique pattern was observed in malate concentration changes: it was highest at 4 mM at 12 AM, decreased to 0.5 mM during the light period, remained very low at about 0.5 mM during the dark period, and then increased during the second light period. These changes were not affected by the night temperatures (Figure 5B and Figure 12B).

Low-molecular-weight organic acids are essential metal-binding ligands that help maintain metal homeostasis, support various metabolic processes, and mediate responses to biotic and abiotic stress [37]. Malate, citrate, and oxalate play crucial roles in metal transport and detoxification in plants. In poplar plants, a multi-omics analysis of xylem sap conducted across different ammonium and nitrate concentrations found that nitrate increased malate levels [38]. However, the addition of nitrate to a culture solution decreases organic acid concentrations in soybean xylem sap, especially malate, compared with control plants grown with an N-free solution [11]. Because malate is a principal energy and carbon source for bacteroids to support nitrogen fixation in soybean nodules [39], a decrease in malate concentration in the xylem sap may be related to the elevated malate consumption in the nodules and roots. Nitrate supply reduces the concentrations of all organic acids in xylem sap [7,11], partly because nitrate acts as a counteranion to transport cations, such as K^+^, Ca^2+^, and Mg^2+^, and partly because organic acids are replaced by nitrate under N-free conditions.

Malate is ubiquitously present in most plant species [40]. This critical metabolite not only supports plant growth through its dual role in the tricarboxylic acid cycle and glycolytic pathway, but also mediates plant–environment interactions by regulating fundamental processes, such as stomatal dynamics, aluminum detoxification, pH homeostasis, and stress adaptation. Malate serves as a key osmoticum in guard cells, where its accumulation regulates turgor pressure to drive stomatal opening. Malate secreted from roots chelates toxic Al^3+^ ions in the rhizosphere to form non-toxic complexes, and malate acts as a biochemical pH stat through its interconversion with citrate in the TCA cycle. Malate maintains redox balance by modulating NAD(P)H/NAD(P)^+^ ratios under oxidative stress.

Vitor et al. [41] reported that a substantial increase in the xylem malate of soybean plants transferred to the N-free medium was derived from aspartate supplied from the shoot via the phloem transport. Supplying ^13^C-aspartate to the intact roots revealed that malate in the xylem sap is readily labelled with ^13^C under N starvation. The results of this research, which show that diurnal changes in malate concentration in xylem sap exhibit a unique pattern, might be due to malate in the xylem derived from feedback aspartate via phloem from the shoot during day-time.

### 3.6. Diurnal Changes in Xylem Sap pH

The xylem sap pH was the lowest at 6.0 at 12 AM, then increased to 6.9 at 10 PM (Figure 6). During the dark period, the pH values were about 6.8. After the second light period, pH decreased to 6.1 at 8 AM. Long-distance signals in the xylem from roots to leaves are essential in plant responses to drought stress. Drought increases the pH of *Commelina communis* xylem sap from 6.1 to 6.7 [42]. The conductance of transpiring leaves is 50% lower in pH 7.0 than in pH 6.0 buffers. Authors have suggested that the increase in apoplastic ABA accumulation at pH 7.0 may result from reduced symplastic sequestration of ABA into guard cells.

### 3.7. Relationships Between the Transport Rate and the Water Flow Rate, and Between the Concentration and the Water Flow Rate

Under both 28 °C day/18 °C night (Figure S1) and 28 °C day/28 °C night (Figure S3) conditions, the transport rates of all the compounds analyzed were positively correlated with the water flow rate. This positive correlation is consistent with the fact that the primary force for transporting these compounds is water flow from the roots to the shoot. No, or a weak correlation was observed between concentration and water flow rate for most compounds (Figure S4), suggesting that changes in water flow rate did not influence the concentrations of major constituents in xylem sap.

### 3.8. Comparison of Diurnal Changes Between Under 28 °C Day/18 °C Night and 28 °C Day/28 °C Night Conditions

The diurnal changes in xylem sap exudation rate and the transpiration rate were similar between the plants under 28 °C day/18 °C night and 28 °C day/28 °C night conditions, indicating that the night temperature changes did not change the water movement from the roots to the shoot, either through the xylem sap exudation rate or transpiration rates. Both the xylem exudation rate and the transpiration rate remained constant during the dark period, although the levels were much lower than in the light period.

The changes in the concentration and transport rate of the major N compounds allanoate, allantoin, Asn, and Gln showed similar patterns under 28 °C day/18 °C night and 28 °C day/28 °C night conditions. This result suggests that night temperature did not have a significant effect on N_2_ fixation, assimilation, and transport processes. On the other hand, the changes in the concentrations of K, Mg, and Ca were different between the plants under 28 °C day/18 °C night and 28 °C day/28 °C night conditions. Under 28 °C day/18 °C night conditions, the concentrations of these cations were constant or relatively lower during the dark period. Still, the concentrations of K and Mg were higher during the dark period under 28 °C day/28 °C night conditions. This result may be due to a decrease in cation absorption rates at low night temperatures (18 °C) compared with high temperatures (28 °C). Both changes in Pi and SO_4_ concentrations were similar between plants under 28 °C day/18 °C night and 28 °C day/28 °C night conditions. Pi concentrations remained constant, but SO_4_ concentrations sharply increased during the dark period. Similarly, changes in citrate and malate concentrations were similar between plants under 28 °C day/18 °C night and 28 °C day/28 °C night conditions. Citrate concentrations increased during the dark period, but malate concentrations sharply declined.

### 3.9. Application for Plant Diagnosis

The method for measuring the transpiration rate of a detached shoot can be used to diagnose nutrient levels in field-grown soybeans. It may also apply to other plant molecules, such as phytohormones, peptides, microRNAs, heavy metals, and agrochemicals, which are transported through the xylem to regulate plant physiology and development. Monitoring the sap traits of grapevines at dormancy can serve as an early diagnostic tool to guide pruning, irrigation, and fertilization, and support the selection of climate-resilient cultivars and rootstock–scion combinations [43].

Transpiration rate can be measured using porometers or infrared gas analyzers, lysimeters that measure intact soil column weight loss, Eddy covariance, and remote sensing methods with satellite image analysis [44,45,46]. Whole-plant chambers linked to an infrared gas analyzer are costly, and replication is time-consuming [47]. Lysimeters often fail to separate soil evaporation from transpiration accurately. Satellite methods and Eddy covariance can capture water dynamics from the field to entire regions. Still, increasing scale implicitly loses resolution and increasingly relies on assumptions to distinguish whole-plant transpiration from evaporation [48].

The transpiration rate of 26 crops was measured using the chamber method, in which the plant was enclosed in a transparent chamber, and the humidity of the inlet and outlet gases was measured [45]. The total amount of transpiration through the whole stage was high, 149 kg/plant, in soybeans compared with other crops. As a result, the water requirement was also high, 584 g water per 1 g dry matter production. Ferrara and Flore [49] compared various methods, including gravimetric analysis, heat-pulse velocity, time-domain reflectometry, and infrared gas-exchange methods, for measuring transpiration in potted apple trees. They concluded that time-domain reflectometry is accurate and not statistically different from the control gravimetric analysis.

The method for measuring transpiration rate from detached shoots that was used in this research required no chambers or equipment and is easy to handle in the field. In this report, we used 30 DAP young plants, so we extended their stems by connecting a tube below the cut stem, because the primary leaves obstructed us from reaching the cut end of the stem with the tube. However, when we used a larger plant, the cut stem was long enough to reach the bottom of the tube [34].

## 4. Materials and Methods

### 4.1. Plant Cultivation

Soybean seeds (*Glycine max* [L.] Merr., cv. Williams) were soaked in 70% ethanol for 30 sec and washed thoroughly with tap water. Then, they were sterilized with 0.5% sodium hypochlorite solution for 5 min and thoroughly washed with tap water. The sterilized seeds were inoculated with a suspension of *Bradyrhizobium diazoefficience* (USDA110) and planted in a vermiculite bed. At 7 DAP (days after planting), plant seedlings were transplanted into 800 mL of nitrogen-free nutrient solution [33] in a 900 mL glass bottle covered with aluminum foil with continuous aeration. Plants were cultivated in a biophotochamber (LH-350S, Nippon Medical & Chemical Instruments Co., Ltd., Osaka, Japan) at 28 °C during the day/18 °C during the night, 55% relative humidity, and a photon flux density of 228 μmol m^−2^ s^−1^ with a 16 h photoperiod and an 8 h dark period. At 9 PM, the lights turned off, and at 5 AM, they turned on. We used temperature and light regimes to promote healthy soybean plant growth in a chamber. The culture solution was renewed every 3 days. Plants at 30 DAP were used for the experiments. The composition of the culture solution was as follows: K_2_HPO_4_ 49 μM, K_2_SO_4_ 626 μM, KCl 12.6 μM, CaCl_2_ 1250 μM, MgSO_4_ 499 μM, Fe-EDTA 50 μM, H_3_BO_3_ 5.94 μM, CuSO_4_ 0.128 μM, MnSO_4_ 0.772 μM, ZnSO_4_ 0.501 μM, CoCl_2_ 0.181 μM, (NH_4_)_6_Mo_7_O_24_ 0.00324 μM, and NiCl_2_ 0.0133 μM. The pH of the culture solution was adjusted to 6.0 with either 0.1 M HCl or 0.1M NaOH. The culture solution was continuously aerated by an air pump at 0.5 L/min and changed every 3 days. Changes in air and culture solution temperatures were monitored every 10 min using a thermal sensor (Ondotori, TR-71ui, T&D, Tokyo, Japan). Figure S5 shows the air and solution temperatures under 28 °C day/18 °C night conditions. The air temperature decreased rapidly from 28 °C to 18 °C, whereas the solution temperature decreased slowly and reached 18 °C about 4 h after the change to night. The increase after 5 AM showed a similar time lag. Figure S5B shows the solution temperature changes under 28 °C day/18 °C night and 28 °C day/28 °C night conditions. The solution temperatures were constant around 28 °C under 28 °C day/28 °C night conditions.

### 4.2. Methods for Xylem Sap Collection and the Measurement of Transpiration Rate

Both experiments, under both the 28 °C day/18 °C night and 28 °C day/28 °C night conditions, started at 10 AM and ended at 3 PM the next day. We use the term “water flow rate” from the intact plant because water flow through the xylem depends on both the root pressure and evapotranspiration. Water flow rates in intact plants were measured by weighing bottles with plants every hour. The weight loss over one hour was the water flow rate. Evaporation from the culture solution in bottles without plants was negligible during the experiment. Three intact plants were measured repeatedly. The xylem sap collection and transpiration rate measurements were carried out every 2 h, from 10 AM on 30 DAP to 2 PM on 31 DAP. At 9 PM on 30 DAP, the light went off, and at 5 AM on 31 DAP, it turned on. The position of the stem at 1 cm below the cotyledonary node was cut with a razor blade, and xylem sap was collected for 1 h in a 1.5 mL plastic tube containing quartz wool [33,34]. The weight of the xylem sap was measured by subtracting the weight of the tube with the quartz wool before and after xylem sap collection. We regarded the mass (g) of xylem sap as the volume of xylem sap (mL), because the specific gravity of xylem sap is approximately 1 due to the low dry matter concentration in xylem sap [50]. A volume of distilled water of 1 mL minus the xylem sap volume was added to the tube to fill the sap to 1 mL, and then the diluted xylem sap was extracted from the quartz wool by sucking the liquid out with an automatic pipette and filtering it through a 0.45 μm membrane filter (Advantec, DISMIC 03CP045AN, Tokyo, Japan). It was then stored at −80 °C until analysis. For the measurement of the transpiration rate from the detached shoot, the end of the cut stem used for collecting xylem sap was recut in tap water in a 10 L bucket, and a 5 cm length Tygon tube with 3 mm ID, 5 mm OD was inserted in water to eliminate air bubbles clogging the water transport. The Tygon tube connected to the stem was placed in a 25 mL plastic tube (Eppendorf Japan, Tokyo, Japan) filled with water in a 10 L bucket, and the excess water was discarded to bring the volume to 20 mL. The stem was fixed to the plastic tube with a urethane form. After wiping the water outside the tube and plants, the weight was measured. The plant was then placed under the same conditions in the same chamber (Figure S6). After 1 h, the weight of the 25 mL tube with the detached plant was measured, and the transpiration rate (mL/h) was calculated from the difference between the pre- and post-incubation weights.

In the experiment under 28 °C day/18 °C night conditions, the water flow rate, calculated as the sum of the xylem sap exudation rate and the transpiration rate from the detached shoot, was compared with that in intact plants. The results showed the same water flow rates between decapitated and intact plants at all times. Therefore, we measured the water flow rate of intact plants to determine the nutrient transport rate in the second experiment under 28 °C day/28 °C night conditions. The nutrient transport rate (μmol/h) was calculated by the concentration of the nutrient (mM) multiplied by the water flow rate (mL) [35]. We confirmed that the major nutrient elements had not been depleted in the culture solution during the treatment period.

### 4.3. Analyses of Concentrations of Mineral Nutrients and N Compounds in Xylem Sap

The concentrations of sulfate, phosphate, allantoate, allantoin, Asn, Mal, and Cit in the xylem sap were analyzed via capillary electrophoresis (7100, Agilent Tech nologies, Inc., Santa Clara, CA, USA) using a fused silica tube (inner diameter: 50 µm; length: 104 cm) and a commercial buffer solution (α-AFQ109, Ohtsuka Electronics Co., Ltd., Osaka, Japan), with an applied voltage of 25 kV. The signals from each component were detected at 400 nm and a reference wavelength of 265 nm [33]. The linear dynamic range is 1 × 10^4^ and the noise level is <50 μAU according to the manual. The linear ranges and the detection limits of cations and anions are presented [51]. The concentrations of inorganic cations in the xylem sap and culture solution were determined using ion chromatography (IC-2010, Tosoh Techno System, Inc., Tokyo, Japan). The maximum range is 5000 mS/cm, and the noise level is 1 nS/cm according to the manual. The linearity range, correlation coefficient, detection limits, and relative standard deviation were reported for this method [52].

An aliquot of xylem sap was used to measure pH with a pH meter (LAQUA twin B-71X, Horiba Ltd., Kyoto, Japan). The pH meter was calibrated at 6.86 and 4.01 using standard pH buffers.

### 4.4. Statistics

The experiments were conducted with three biological replications. The plants were cultivated using a random arrangement in a growth chamber. Statistical significance between the average values of the 1st light period (*n* = 18; 6 time points and 3 replications) and the dark period *n* = 12; (*N* = 18; 6 time points and 3 replications), and between the dark period and the 2nd light period (*n* = 15; 5 time points and 3 replications), was analyzed using EXCEL software. Individual data on the xylem sap exudation rate, the transpiration rate of the detached shoot, and the concentrations and the transport rate of constituents were analyzed by Student’s *t*-test; independent samples, two-tailed, significance level *p* = 0.05. Individual data on the water flow rate in intact plants were analyzed using Welch’s *t*-test. Statistical significance between the average values of the 1st light period (*n* = 36; 12 time points and 3 replications) and the dark period (*n* = 24; 8 time points and 3 replications), and between the dark period and the 2nd light period (*n* = 30; 10 time points and 3 replications), was analyzed using EXCEL software. Correlations between the average water flow rate and the average transport rate and between the average water flow rate and the average concentration of each component (*n* = 15) were analyzed by Pearson’s correlation coefficient (r).

## 5. Conclusions

The diurnal changes in the sum of the xylem sap exudation rate and transpiration rate of the detached soybean shoot were similar to the water flow rate of the intact plants, supporting the idea that the transpiration rate of soybean plants in the field can be estimated using the sum of the xylem sap exudation rate and transpiration rate of the detached shoot. Both the xylem sap exudation rate and transpiration rate were much lower under dark conditions than under light conditions, but were not zero under dark conditions. All the N compounds showed similar patterns, with higher concentrations and lower transport rates during the dark period than during the light period. The transport rates of the primary N compounds, allantoate, allantoin, and Asn, increased during the light period, reaching a maximum around 2 PM, then decreased. During the dark period, the transport rates of these compounds were lower than during the light period, despite their higher concentrations. The proportions of allantoate, allantoin, Asn, and Gln were constant throughout the day and night periods. When comparing the low night temperature of 18 °C and the high night temperature of 28 °C, the xylem sap compositions and transport rates of N compounds, anions, and organic acids were not affected. Still, those of K and Mg differed between the low- and high-night-temperature regimes. This experiment used only one soybean variety, so further experiments are required to evaluate the universality of the trends observed in Williams.

## Data Availability

The raw data supporting the conclusions of this article will be made available by the authors on request.

## Supplementary Materials

The following supporting information can be downloaded at: https://www.mdpi.com/article/10.3390/plants15040561/s1, Figure S1. Relationships between the water flow rate and the transport rate of each constituent of soybean plants under 28 °C day/18 °C night conditions. r: Pearson’s correlation coefficient. Figure S2. Relationships between the water flow rate and the concentration of each constituent of soybean plants under 28 °C day/18 °C night conditions. r: Pearson’s correlation coefficient. Figure S3. Relationships between the water flow rate and the transport rate of each constituent of soybean plants under 28 °C day/28 °C night conditions. r: Pearson’s correlation coefficient. Figure S4. Relationships between the water flow rate and the concentration of each constituent of soybean plants under 28 °C day/28 °C night conditions. r: Pearson’s correlation coefficient. Figure S5. Temperature changes in the air in the growth chamber and the culture solution under the controlled conditions. (A) Air temperature and the culture solution temperature under 28 °C day/18 °C night conditions. (B) Comparison of the solution temperatures between under 28 °C day/18 °C night and under 28 °C day/28 °C Night conditions. The grey background shows night-time from 9 PM to 5 AM, and the white background shows day-time from 5 AM to 9 PM. Figure S6. The photograph of measuring the transpiration rate from the detached shoot.

## Abbreviations

The following abbreviations are used in this manuscript:

Gln	Glutamine
Asn	Asparagine
DAP	Days after planting
