# Supplementary figures and images for: Diurnal Changes in the Transport Rates of Ureides, Amides, Cations, Anions, and Organic Acids Estimated by Xylem Sap Exudate and the Water Flow Rate of Soybean Plants
Source: Plants (Basel). 2026 Feb 11;15(4):561. doi: 10.3390/plants15040561 (PMC12944621; doi:10.3390/plants15040561)

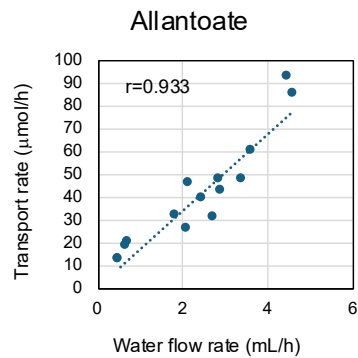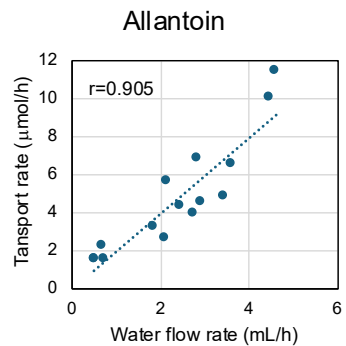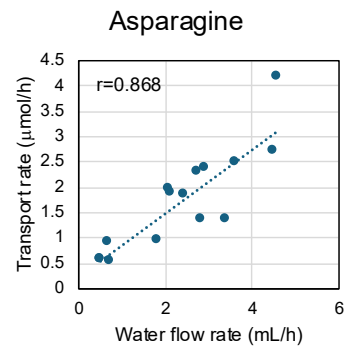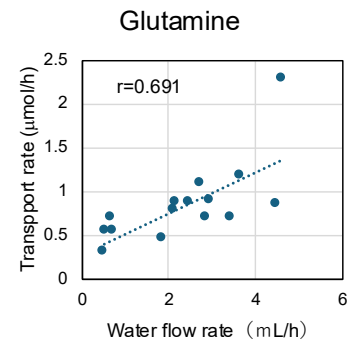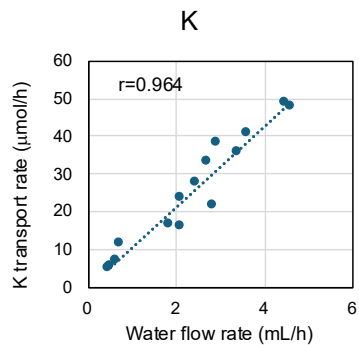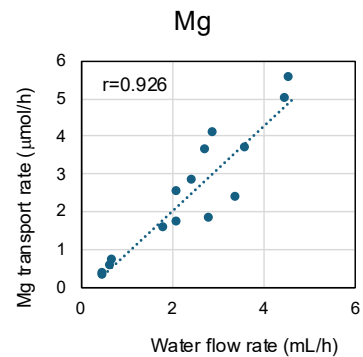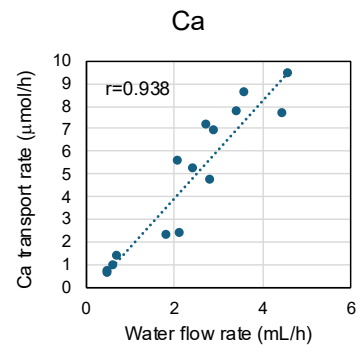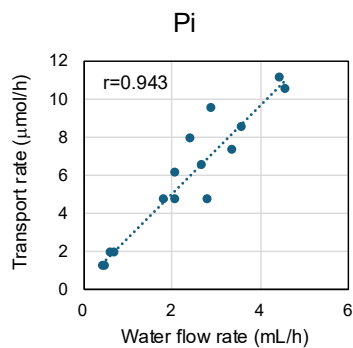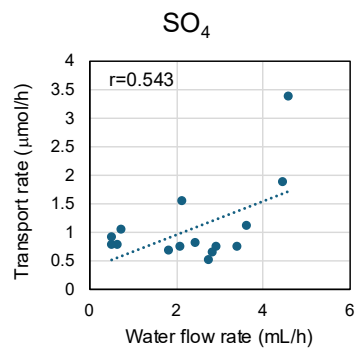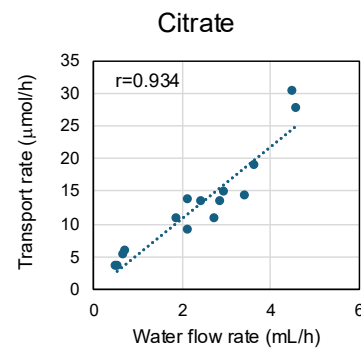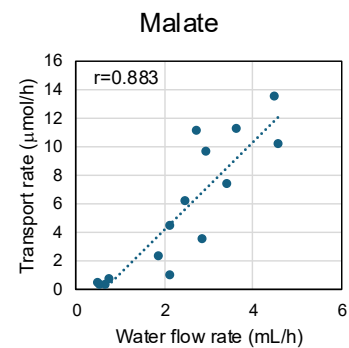

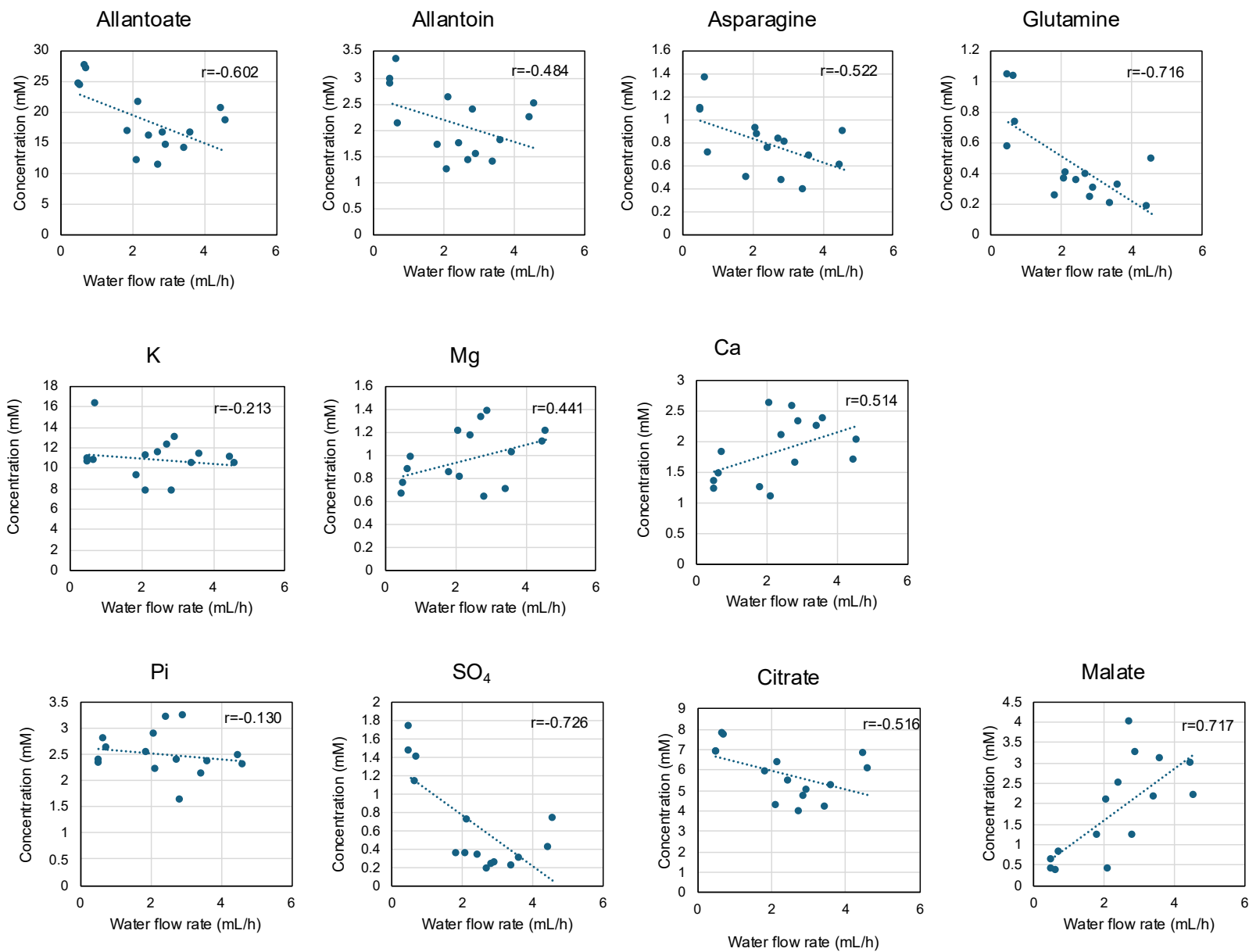

Figure S2

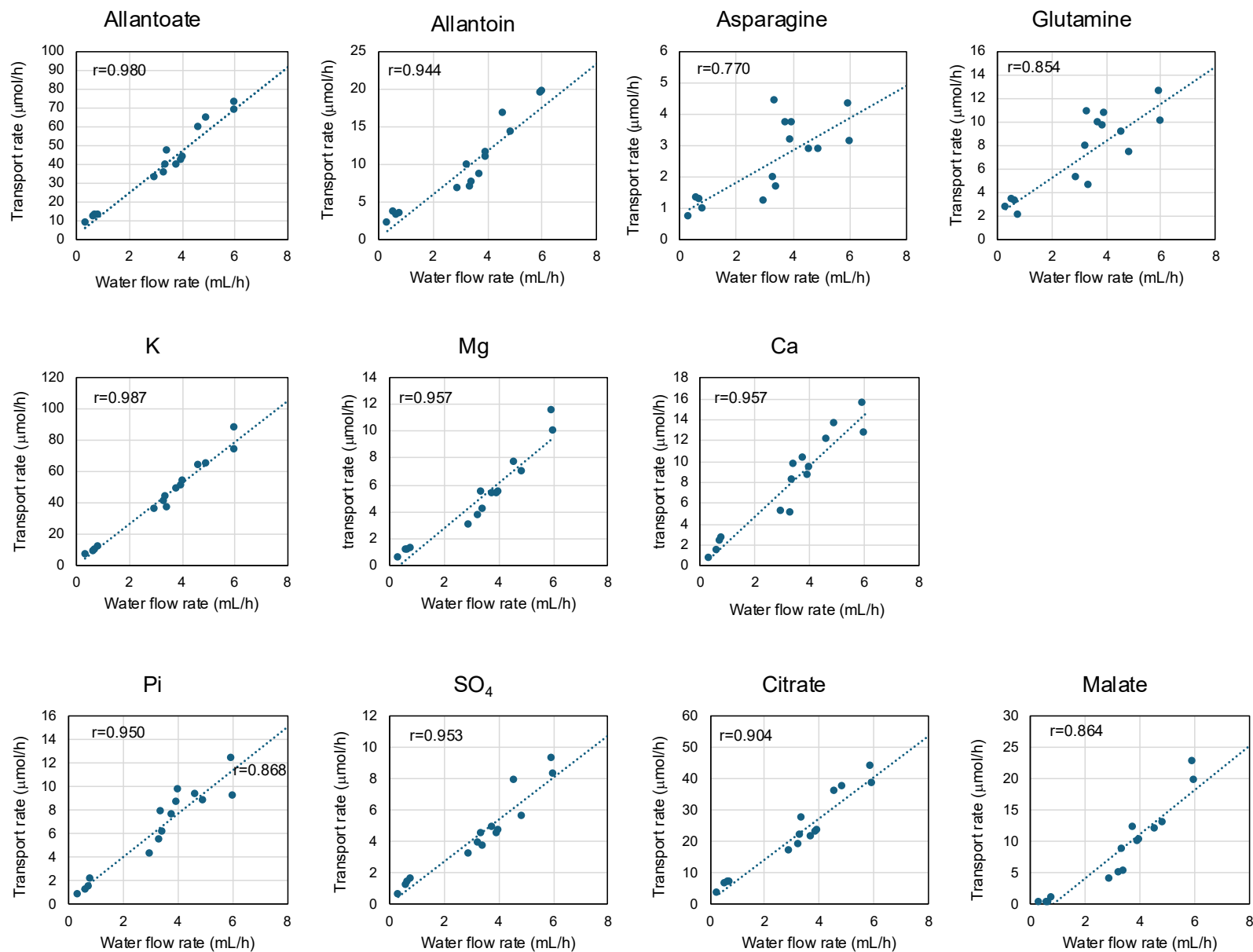

Figure S3

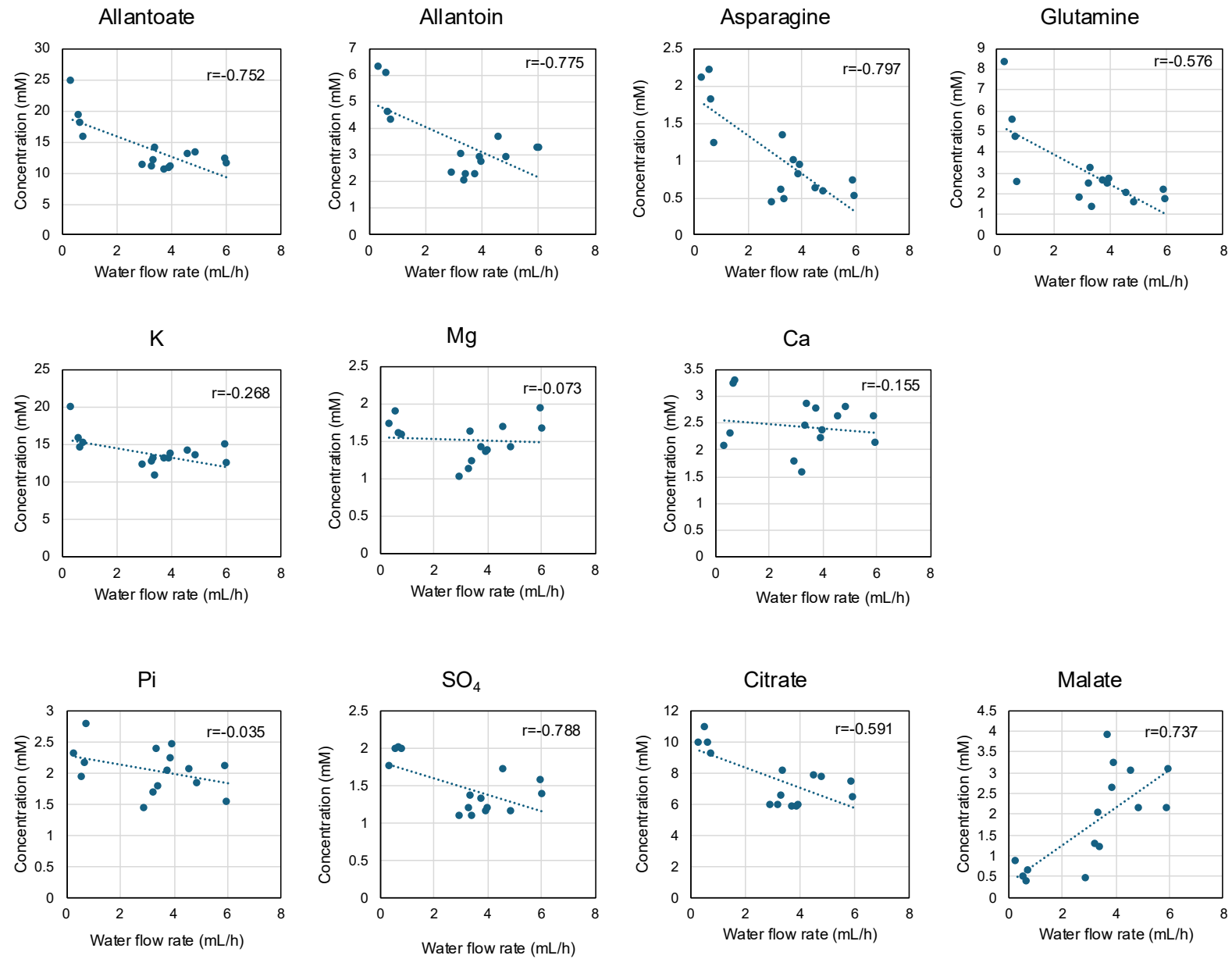

Figure S4

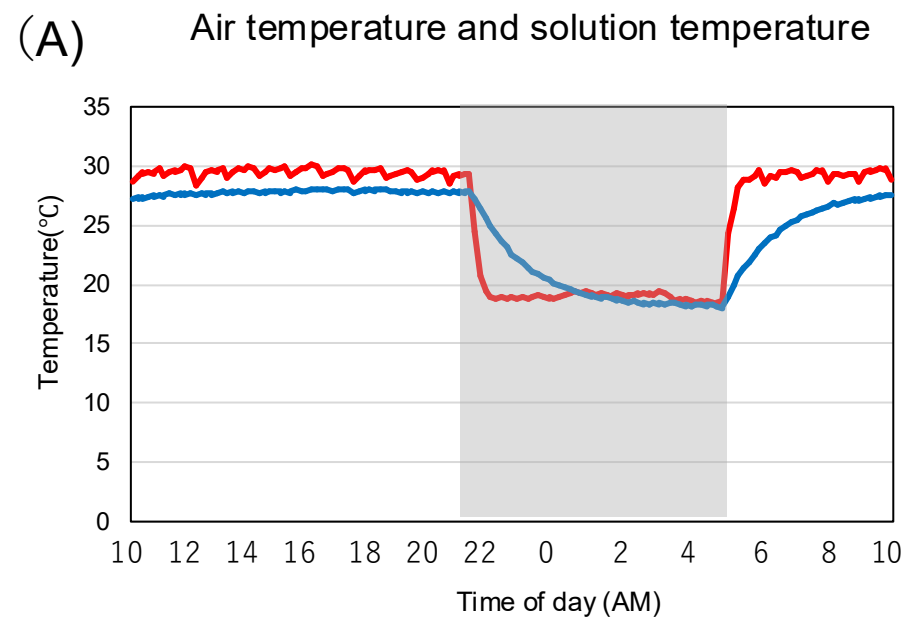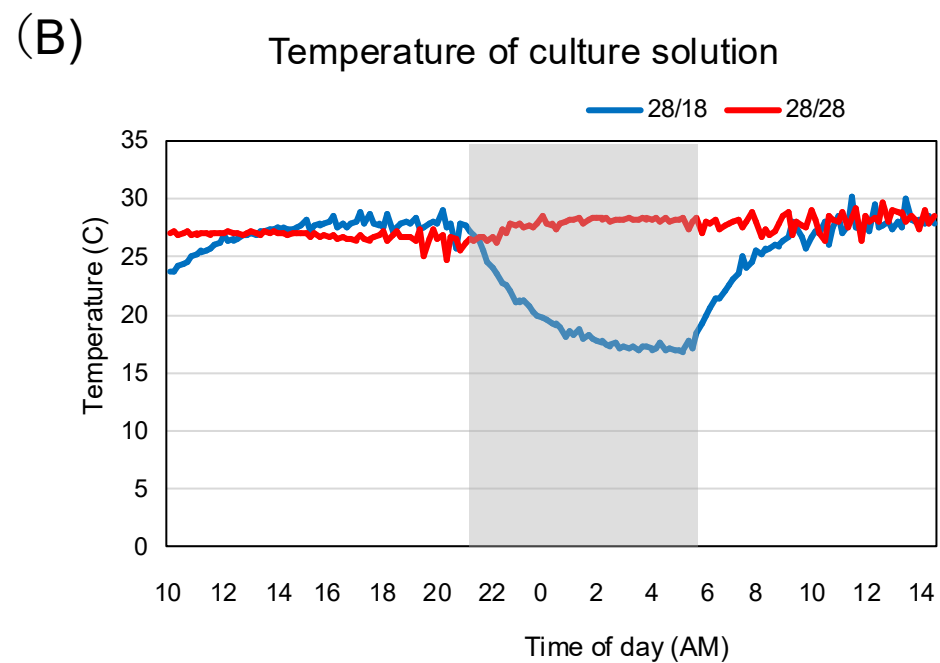

Figure S5

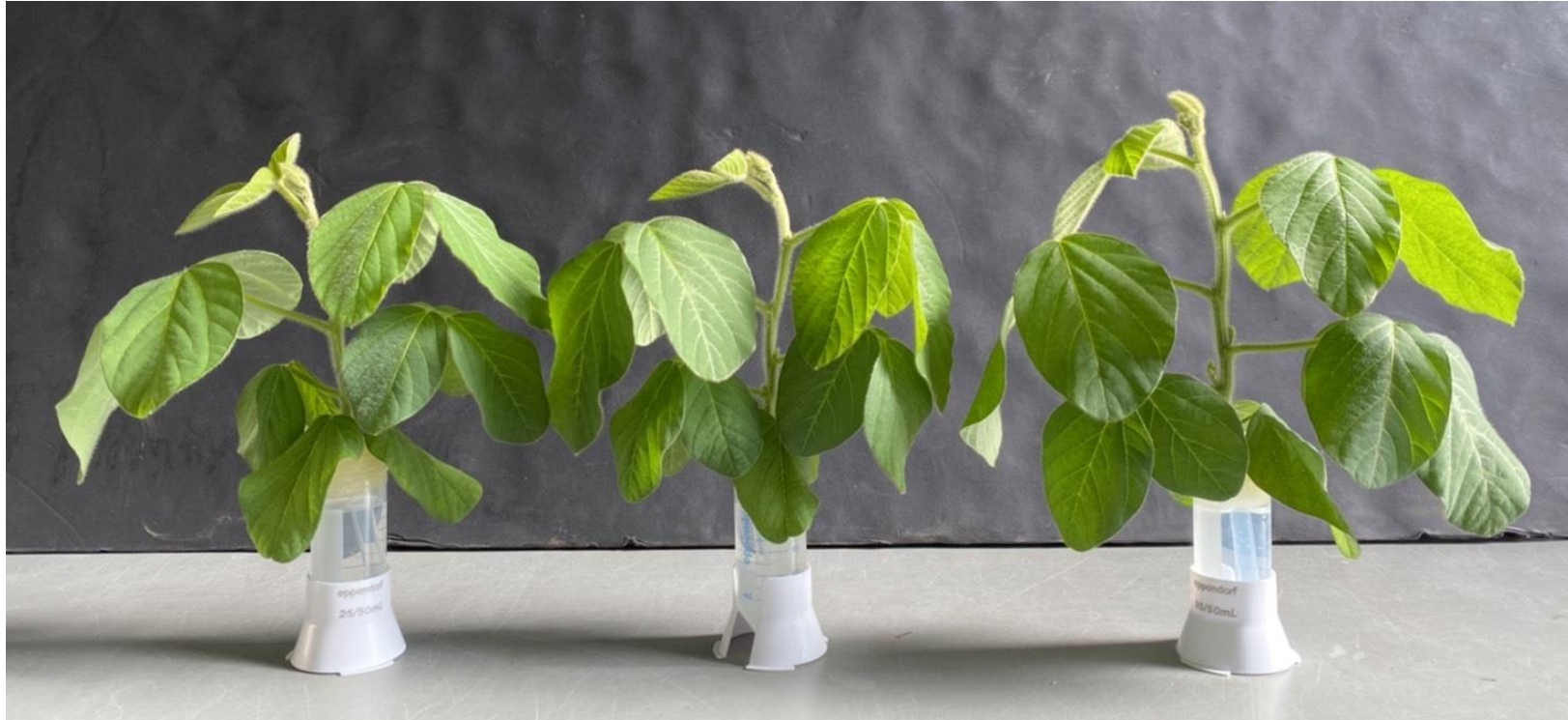

Figure S6

Supplement: Supplementary file 1 [file plants-15-00561-s001.zip › plants-4099931-supplementary.pdf]
